# Vertical bone regeneration with a simultaneous dental implant in the aesthetic zone

**DOI:** 10.21142/2523-2754-1002-2022-112

**Published:** 2022-06-27

**Authors:** Fabio Andrés Jiménez Castellanos, Paola Andrea Castro Pereira, Wilson Orlando Peña Pineda

**Affiliations:** 1 Institución Universitaria Colegios de Colombia UNICOC, Facultad de OdontologíaUniversidad Antonio Nariño UAN. Bogotá, Colombia.fjimenezc@unicoc.edu.co Universidad Antonio Nariño Institución Universitaria Colegios de Colombia UNICOC Facultad de Odontología Universidad Antonio Nariño UAN Bogotá Colombia fjimenezc@unicoc.edu.co; 2 Práctica privada. paola.cp01@gmail.com, wpena45@uan.edu.co Práctica privada paola.cp01@gmail.com wpena45@uan.edu.co

**Keywords:** dental implants, bone regeneration, reconstructive surgical procedures, aesthetics, dental procedures, implante dental, regeneración ósea, procedimientos quirúrgicos reconstructivos, estética, dental

## Abstract

**Introduction:**

Bone defects hinder implant positioning. Vertical bone deficiency is the most challenging for clinical treatment, due to the high sensitivity of the technique and frequent intra- and postoperative complications. An alternative treatment for vertical defects is guided bone regeneration with simultaneous implantation; however, few studies have evaluated its effectiveness over time.

**Objective:**

To evaluate peri-implant tissue stability in a dental implant simultaneously positioned in an aesthetic zone of an area of zone vertical bone regeneration 3 years after functional load.

**Case Presentation:**

A 62-year-old male presented with the absence of the right lateral incisor and vertical bone defect of 3mm. His initial condition affected the relation of zenith points, causing disharmony. After case evaluation, vertical guided bone regeneration and simultaneous dental implants were performed to obtain a vertical bone gain of 3mm. The implant was rehabilitated with a zirconium crown and evaluated 3 years after functional loading.

**Conclusion:**

Vertical guided bone regeneration and simultaneous dental implant in the aesthetic zone in small bone defects (<4 mm) is an appropriate surgical and prosthetic technique to reduce surgical time, providing stability of peri-implant tissues even 3 years after functional implant loading.

## RESUMEN

**Introducción:**

La presencia de defectos óseos dificulta el posicionamiento de los implantes, y es la deficiencia ósea vertical la que representa un mayor reto para el clínico por la alta sensibilidad de la técnica y las frecuentes complicaciones intra y posoperatorias. Una alternativa de tratamiento para los defectos verticales es la regeneración ósea guiada con implante simultáneo; sin embargo, en la literatura existen pocos estudios que evalúen su efectividad en el tiempo.

**Objetivo:**

Evaluar la estabilidad de los tejidos periimplantares en un implante dental posicionado simultáneo a la regeneración ósea vertical en una zona estética, luego de 3 años de carga funcional.

**Presentación de caso:**

Paciente masculino de 62 años que presenta ausencia del incisivo lateral derecho con un defecto óseo vertical de 3 mm, que crea una desarmonía de los puntos cenit. Posteriormente a la adecuada evaluación del caso, se decide realizar una regeneración ósea guiada vertical y colocar un implante dental simultáneo, y se obtuvo una ganancia ósea vertical de 3 mm. El implante es rehabilitado con corona en zirconio y evaluado luego de 3 años de la carga funcional.

**Conclusión:**

La regeneración ósea guiada vertical y el implante dental simultáneo en zona estética en defectos óseos pequeños (<4 mm) es una técnica quirúrgica y protésica adecuada para reducir los momentos quirúrgicos, ya que proporciona estabilidad a los tejidos periimplantares, luego de 3 años de la carga funcional del implante.

## INTRODUCTION

Implant therapy is currently an option for prosthetic rehabilitation of edentulous areas that simulate natural teeth function and aesthetics. However, in some cases, horizontal and vertical bone deficiencies may occur due to different events, such as fractures, and endodontic or periodontal problems. Within this scenario, the complexity of dental implants increases. Bone defects pose the most significant challenge due to the high sensitivity of the technique and frequent intra- and postoperative complications. ^1^

Different alternatives for vertical bone augmentation, such as guided bone regeneration (GBR), onlay bone-block grafting (OBG), and distraction osteogenesis (DO), have been reported in the literature. Autologous OBGs was considered the "gold standard" for severe atrophies; however, advances in bone graft biomaterials favor less invasive approaches such as GBR. GBR is a predictable technique that has been broadly implemented after the development of guided tissue regeneration, based on the principle of using the same epithelial cells and connective tissue exclusion to favor bone formation. ^2^

Vertical GBR with simultaneous dental implant positioning has also undergone some developments. This technique reduces surgical time and generates greater patient satisfaction with a reduction of treatment time. ^3^ This technique is, however, more challenging given its nature. An implant platform should be used as a stabilizer while lifting the membrane to a curved-shaped position as it does not have retaining walls. The purpose of this procedure is to comply with the principles for predictable bone regeneration (PASS) : 1) primary wound closure, 2) angiogenesis, 3) space creation and maintenance, and 4) wound stability to include blood clot formation and bone graft. ^4^

On review of the the literature, it was observed that most studies focused on GBR before implant positioning, with only a few evaluating simultaneous techniques. ^5^^,^^6^ Nonetheless, this simultaneous technique has caused some controversy due to its unpredictable behavior over time. Hence, this case report aims to evaluate peri-implant tissue stability in a dental implant simultaneously positioned in an aesthetic zone of a vertical bone regeneration area 3 years after functional loading. 

## CASE PRESENTATION

**Fig. 1:**
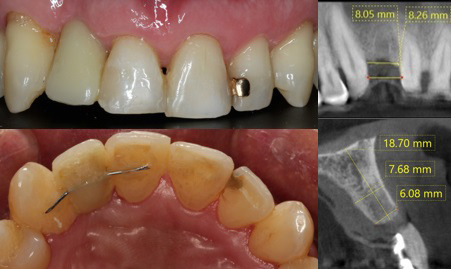
Initial clinical and tomographic analysis.

A 62-year-old male patient with no ongoing systemic personal history attended a consultation with the Periodontics Postgraduate Program at the *Institución Universitaria Colegios de Colombia* in September 2016, referred by the Oral Rehabilitation Postgraduate Program to rehabilitate the edentulous area of the right lateral incisor with an implant-supported restoration. Extra and intraoral tests, together with diagnostic imaging evaluation and complete medical history were performed. A vertical defect of 3mm was evidenced in tooth 12 (Fig. 1). Clinical examination showed infectious foci, active periodontal disease, and dental caries. Subsequently, alginate dental impressions were taken, and plaster models were made for later articulator mounting and occlusion analysis.

**Fig.2. A:**
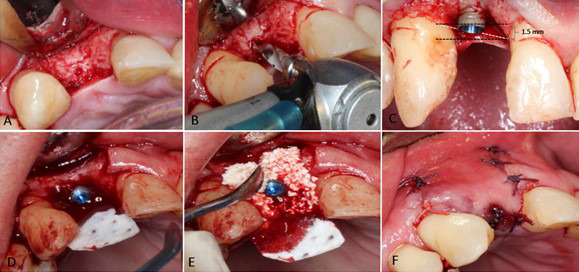
Flap B. Surgical preparation. C. Dental implant positioning. D. Collagen membrane E. Bone graft F. Suture.

Following thorough analysis of the case, the patient underwent the hygienic phase and was later asked to approve the performance of vertical GBR while simultaneously positioning the implant. This procedure was suggested to correct the vertical defect in the area and modify the zenith point concerning the other teeth in the aesthetic area. The patient accepted the suggested treatment and provided signed informed consent. The surgical procedure was performed in September 2016, starting with asepsis, antisepsis, infiltrative anesthesia with lidocaine 2% (3 carpules in total), followed by paracrestal incision with a 15c scalpel blade on the edentulous area of the right lateral incisor. Afterwards, a vertical incision mesial and distal to 12 and aided by the buser periosteal elevator allowed reflecting the flap at full-thickness (Fig. 2-A). Subsequently, the milling protocol recommended by the manufacturer for bone type III was initiated to provide primary stability to the dental implant (Fig. 2-B); milling was performed with abundant irrigation of physiological saline. Parallelism was verified, and the 3.3mm x 10 mm V3 implant by miss implants was positioned at 30N torque without irrigation. The implant was positioned supra-crest at 3 mm, that is, at the level of the mesial bone crest of tooth 13 and 1.5 mm apical to the distal bone crest of tooth 11 (Fig. 2-C).

Vertical GBR was completed after implantation. It was initiated by stabilizing a collagen membrane in palatine (Fig. 2-D), then 0.5cc of bovine xenograft was positioned around the implant (Fig. 2-E). The GBR was completed by leaving the membrane to rest over the implant, creating a curved-shaped position. The membrane was stabilized with reabsorbable stitches 5-0. Finally, the flap was coronally positioned with simple stitches and reabsorbable suture 5-0 (see Fig. 2-F). At the end of the procedure, the patient received some recommendations. Further treatment was initiated with amoxicillin 500 mg, every 8 hours for 8 days, and ibuprofen 800 mg ,every 12 hours for 3 days.

**Fig. 3:**
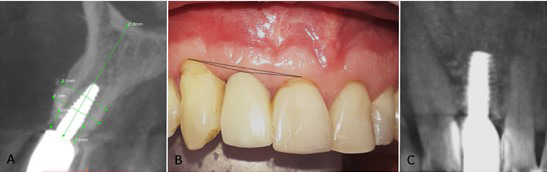
A and C Final tomography. B. Clinical photo 3 years post-functional loading.

Five months later (February 2017), the second surgical phase was performed using a roll technique to increase vestibular gingival thickness. The provisional implant was functional throughout the whole integral rehabilitation process. In October 2017 (13 months after initial positioning), the abutment was torqued to 20N, and the final crown was cemented in zirconium. Follow-up appointments were scheduled every 6 months. The peri-implant tissues were evaluated in October 2020 (3 years after functional loading) (Fig. 3).

The patient reported satisfaction with the treatment results, in which the initial vertical bone defect in the edentulous area of tooth 12 was corrected. As a result, an average vertical gain of 3 mm with support of the bony ridges of the adjacent teeth moderately correcting the zenith point of the zone. Likewise, the roll technique performed in the second surgical phase produced adequate vestibular gingival thickness. These results have remained stable over the 3-year follow-up period.

## DISCUSSION

The selection of the most adequate surgical technique in each clinical case must be carefully considered. Different aspects such as the edentulous section, residual bone tissue volume, proximity to vital structures, soft tissue coverage, and the systemic condition of the patient and the skills and preferences of the surgeon must always be taken into account. ^7^

According to the decision tree for vertical bone augmentation by Plonka et al., the technique must be chosen according to the type of bone defect. ^8^ They recommend GBR for small vertical bone defects (< 4 mm) and suggest considering simultaneous dental implant positioning when an average vertical gain of 3 mm is desired. The OBG technique contributes to a higher average vertical gain (4.75 mm) albeit with an increase in the presentation of complications compared to GBR. ^8^ Urban et al. reported that the most complicated technique is DO (47.3%), followed by OBG (23.9%) and, finally, GBR (12.1%). ^9^ Therefore, according to the decision tree, the vertical GBR technique with simultaneous implant positioning was chosen for the present case, since it was necessary to gain between 3 and 4 mm in height. 

Barrier membranes are used in the GBR technique, and are useful for maintaining space, stabilizing blood clots, and excluding non-bone-forming cells. ^4^ Bone formation starts within the first 24 hours, but this new formation only becomes visible during the fourth week after GBR, depending on the angiogenesis present. Therefore, barrier membranes are essential to achieve the objectives of GBR. ^10^ There are reabsorbable and non-reabsorbable membranes. The latter were initially used considering their mechanical properties; however, they require a second intervention and have a high exposure rate (30-40%), which may cause more significant complications. On the other hand, reabsorbable membranes, mainly collagen, have a three-dimensional porous semi-permeable structure that contributes to the transfer of nutrients, angiogenesis, hemostasis, early clot stability, and fibroblast chemotaxis. However, these membranes also causes rapid biodegradation due to the enzymatic activity of macrophages, polymorphonuclear leukocytes, and periodontal bacteria. ^11^

Urban et al. recently described an average vertical gain in GBR with 3.51 mm (*n* = 7; 95% confidence interval [CI] 2.80-4.22; *p* < 0.001) of reabsorbable membrane, compared to the mean vertical gain of 4.42 mm with non-resorbable membrane (*n* = 13; 95% CI 3.97-4.87; *p* < 0.001). ^9^ In addition, they reported that reabsorbable membranes were more prone to complications than non-resorbable membranes (23% vs. 7%) regardless of the time of implant positioning. Urban et al. suggest that these differences may be due to careful handling of non-reabsorbable membranes by the surgeon since complications linked to these membranes are more difficult to manage. ^9^ In turn, Plonka et al. recommended the use of the two types of membrane for small vertical bone defects (< 4 mm). ^8^ Hence, it is important to highlight that either membrane can be used with favorable results for small vertical bone defects.

In addition to the use of barrier membranes, bone substitutes such as autografts, allografts, xenografts, and alloplastics are also used in GBR to increase bone volume. Autologous bone is considered the gold standard due to its osteogenic, osteoinductive, and osteoconductive properties: However, it is associated with a rapid reabsorption rate and donor site morbidity. Moreover, it is limited by the size and shape of the graft. ^12^ A xenograft is a bone substitute obtained from a species other than humans (mainly animals), the most common being of bovine, porcine, and equine-origin. Xenografts are biocompatible and have osteoconductive properties that favor angiogenesis, migration, and cell differentiation. In the long term, native bone formation is observable among the particles of this kind of graft. Additionally, these grafts are commercially available and do not transmit diseases due to sterilization protocols. To date, the xenograft with the greatest significant scientific support in the literature is the mineralized bovine origin xenograft that has been tested in vitro and in vivo. These xenografts have slow reabsorption and human-bone-tissue-like surface porosity and chemical composition. Similarly, they work as a mineral scaffold by having a three-dimensional pore network that allows rapid penetration and absorption of blood and serum proteins. ^13^ Fontana et al. reported that vertical GBR with a xenograft and autograft combination and non-reabsorbable membrane presented a mean vertical bone gain of 4.14 ± 1.33 mm and a 1-6-year follow-up implant survival of 93.6% and a vertical GBR success rate plus simultaneous implant placement of 82.5%.^14^ In contrast, Guarnieri et al. reported that, the use of reabsorbable membrane and xenograft in vertical GBR achieved an average vertical bone gain of 3.3 mm. ^15^ In the present case, a xenograft was used in combination with a collagen membrane, obtaining an average vertical bone gain of 3 mm.

It is important to note that there may be a percentage of bone reabsorption after surgery with bone augmentation procedures. Urban et al. reported that the amount of marginal bone loss would be impacted by the type of surgical procedure, describing bone loss for DO at 1 year of 1.4 mm (*n* = 1; weighted mean difference [WMD] = 1.40 mm; 95% CI 1.33-1.47; *p* < 0.001) and 0.58 mm for GBR with reabsorbable membrane (*n* = 1; WMD = 0.58 mm; 95% CI 0.19- 0.97; *p* < 0.001). ^9^ In our case, however, no marginal bone loss was observed after 3 years of post-load follow-up.

## CONCLUSION

Based on this case report, it can be concluded that vertically guided bone regeneration and simultaneous dental implant in the aesthetic zone with small bone defects (<4 mm) is an appropriate surgical and prosthetic technique to reduce surgical time. This technique also provides the stability of peri-implant tissues 3 years after functional implant loading. Nonetheless, thorough case analysis must be performed for selection of the most adequate technique and the recommendations of the literature should be closely followed.

## Financing Sources:

Self-financed
